# Inhibition of TIM‐4 protects against cerebral ischaemia‐reperfusion injury

**DOI:** 10.1111/jcmm.14754

**Published:** 2019-11-27

**Authors:** Lifang Zheng, Yongqian Huang, Xinghua Wang, Xijia Wang, Wei Chen, Wei Cheng, Chunlian Pan

**Affiliations:** ^1^ Department of Neurology The Seventh People's Hospital of Shenzhen Shenzhen China; ^2^ Department of Neurology Puren Hospital Affiliated to Wuhan University of Science and Technology Wuhan China; ^3^ Cancer Institute of Integrated traditional Chinese and Western Medicine Key Laboratory of Cancer Prevention and Therapy Combining Traditional Chinese and Western Medicine Zhejiang Academy of Traditional Chinese Medicine Hangzhou China

**Keywords:** cerebral ischaemia‐reperfusion injury, co‐culture, TIM‐4

## Abstract

TIM‐4 plays an important role in ischaemia‐reperfusion injury of liver and kidney; however, the effects of TIM‐4 on cerebral ischaemia‐reperfusion injury (IRI) are unknown. The purpose of the present study was to investigate the potential role of TIM‐4 in experimental brain ischaemia‐reperfusion injury. In this study, cerebral ischaemia reperfusion was induced by transient middle cerebral artery occlusion (MCAO) for 1 hour in C57/BL6 mice. The TIM‐4 expression was detected in vivo or vitro by real‐time quantitative polymerase chain reaction, Western blot and flow cytometric analysis. In vivo, the administration of anti‐TIM‐4 antibodies significantly suppressed apoptosis, inhibited inflammatory cells and enhanced anti‐inflammatory responses. In vitro, activated microglia exhibited reduced cellular proliferation and induced IRI injury when co‐cultured with neurons; these effects were inhibited by anti‐TIM‐4 antibody treatment. Similarly, microglia transfected with TIM‐4 siRNA and stimulated by LPS + IFN‐γ alleviated the TIM‐4‐mediated damage to neurons. Collectively, our data indicate that the inhibition of TIM‐4 can improve the inflammatory response and exerts a protective effect in cerebral ischaemia‐reperfusion injury.

## 1 INTRODUCTION

As society continues to age, the incidence rate of ischaemic stroke has increased each year and tends to occur even more frequently in younger people.^1^ Iscahemia stroke results from an occlusion of the major cerebral artery and its branches. Vascular occlusion often leads to oxygen and energy deprivation, the formation of reactive oxygen species, disturbed ion balance and provocation of inflammatory processes.^2^ Early cerebral reperfusion with a tissue plasminogen activator (tPA) or mechanical thrombectomy are the conventional therapeutic strategies used to treat acute ischaemic stroke within the appropriate time window.^3^ However, this effective time window for treatment is limited and been shown to be largely beneficial with number needed to treat 10 for intravenous thrombolysis and only 2.6 for mechanical thrombectomy.^4^ Since the majority of patients still come outside the treatment window, there is a need to search for alternative treatment strategies. Recently, several studies reported that immunity and inflammation are involved in the pathogenesis of ischaemic stroke.^5^, ^6^ This evidence implies us that a better understanding of potential molecular mechanisms of immunity in ischaemic stroke would enable the development of targeted approaches to protect against ischaemic injury.

The T cell immunoglobulin and mucin domain (TIM) family consists of eight members (TIM1‐TIM8) in mice and three members (TIM1, TIM3 and TIM4) in humans.^7^ All TIM family protein members are type I cell surface glycoproteins, with each containing a common immunoglobulin V‐like domain, mucin‐like domain, transmembrane domain and a cytoplasmic region.^8^ TIM1, TIM3 and TIM4 have various functions in the immune response and are expressed by different immune cells. TIM‐1 was found to be expressed on activated Th2 cells, whereas TIM‐4 is not expressed on T cells but is primarily found on antigen‐presenting cells (APCs) (ie macrophages and dendritic cells).^9^ Previous studies have implicated TIMs in the regulation of certain immune responses, including allergy, asthma, autoimmunity and transplant tolerance.^8^, ^10^ Recent studies suggest that the TIM‐4 pathway plays an important role in IRI of the liver and kidney. Moreover, decreased TIM‐4 expression has been shown to alleviate IRI under hepatic ischaemic preconditions.^11^ In addition, although the TIM‐1: TIM‐4 pathway has been found to enhance renal IRI,^12^ the effects of TIM‐4 on cerebral IRI remain unknown. Therefore, we hypothesized that TIM‐4 might participate in cerebral IRI. Here, the purpose of the present study was to detect the association between TIM‐4 and ischaemic stroke and the effect of TIM‐4 on ischaemic stroke.

## 2 MATERIALS AND METHODS

### 2.1 Animals

Six‐week‐old male C57BL/6J mice (20‐25 g) were purchased from the Experimental Animal Center of Zhejiang Academy of Traditional Chinese Medicine. All mice were housed in an environmentally controlled room under a 12 hours light/dark cycle with free access to food and water. All animal experiment protocols were approved by the animal committee at Zhejiang Academy of Traditional Chinese Medicine and performed in accordance with the National Institutes of Health Guide for Care and Use of Laboratory Animals (NIH Publications, No. 8023, revised in 1978).

### 2.2 In vivo experiments

#### 2.2.1 Mouse model of middle cerebral artery occlusion (MCAO)

A total of 30 mice were randomly divided into three groups (n = 10): (a) blank group, (b) MCAO group and (c) TIM‐4 mAb + MCAO group. The blank group received the same surgical procedures as the other groups without an occlusion of the carotid. The mice in both the MCAO group and the TIM‐4 mAb + MCAO group were injected with physiological saline and anti‐TIM‐4 antibodies (0.5 mg/kg) at 1 hour prior to the induction of ischaemia. Mouse models of MCAO were established and assessed according to a previous method.^13^ Briefly, the animals were injected with intraperitoneal anaesthesia (4% chloral hydrate). The left common, internal and external carotid arteries were separated sequentially from the left lateral approach to the neck. A silicone cord was inserted from the common carotid artery to the middle cerebral artery. After 60 minutes of embolization, the cord was removed and ligated. After the neurobehavioral credits were assessed according to time, the animals were anaesthetized and sacrificed.

#### 2.2.2 2,3,5‐triphenyltetrazolium chloride (TTC) staining

The mice were sacrificed after 24 hours or 48 hours of reperfusion, and the brains were rapidly removed. Subsequently, 1 mm‐thick coronal sections from each group were used to perform TTC staining. The slices were immersed in a 0.05% 2,3,5‐triphenyltetrazolium chloride (TTC) solution for 30 minutes at 37°C. The total area of each brain section and the infarcted region were quantified using the software program Image J software (v1.46; National Institutes of Health). The infarct volume was corrected for oedema.

#### 2.2.3 Assessment of neurological function

The injury severity of the treated mice was assessed with a modified Bederson score as previously described.^14^ The modified Bederson scores were as follows: 0, no deficit; 1, loss of forelimb flexion; 2, same as 1, plus decreased resistance to lateral push; 3, indicated unidirectional circling; 4, longitudinal spinning or seizure activity; and 5, no movement.

#### 2.2.4 TdT‐mediated biotin‐16‐dUTP nick‐end labelling assay

A TdT‐mediated biotin‐16‐dUTP nick‐end labelling (TUNEL) assay was performed using a One‐Step TUNEL Apoptosis Assay kit (Roche) to detect apoptotic cells in the mouse livers. In brief, 4‐μm‐thick paraffin sections were deparaffinized, hydrated, treated with proteinase K for 20 minutes and subsequently incubated with a mixture of a fluorescent labelling solution consisting of dUTP and the TdT enzyme at 37°C for 1 hour in a humidified atmosphere. As a positive control, the sections were incubated in DNaseI for 10 minutes at room temperature (25°C) before performing the fluorescent labelling procedure. Negative controls were incubated with dUTP for 10 minutes at room temperature (25°C). Subsequently, the samples were treated with diaminobenzidine, counterstained with haematoxylin (to identify the cell nuclei), dehydrated in a gradient series, vitrified with dimethylbenzene and finally mounted on slides with neutral balsam.

#### 2.2.5 Immunohistochemical analysis

The mouse brain tissues were fixed in a 10% formalin solution for 24 hours, embedded in paraffin, sliced, dewaxed and hydrated according to conventional techniques. The samples were then incubated in a 5% foetal bovine serum for 30 minutes at room temperature followed by an overnight incubation at 4°C with the primary antibodies (CD3; ab16669; Abcam; CD68; ab125212; Abcam). The samples were again incubated for 30 minutes at 37°C after the addition of a horseradish peroxidase‐conjugated secondary antibody (anti‐rabbit‐HPR; 7074; Cell Signalling Technology). The samples were then treated with diaminobenzidine for colouration. The cell nuclei were counterstained with haematoxylin and the samples were dehydrated in a gradient series, vitrified with dimethylbenzene and finally mounted on slides with neutral balsam.

#### 2.2.6 Flow cytometric analysis

Peripheral blood mononuclear cells (PBMCs) were isolated by centrifugation on a Ficoll (Lymphoprep; PAA; Nycomed) gradient from buffy coat preparations obtained from mice and then washed twice in ice‐cold PBS. Monocytes were purified from PBMCs using anti‐CD14 magnetic beads (Miltenyi Biotec). The cells were resuspended to approximately 1‐5 × 106 cells/mL in ice‐cold PBS. Next, 100 μL of the cell suspension was added to each tube. The cells were then incubated with 0.1‐10 μg/mL of primary anti‐TIM4 antibodies (ab47637; Abcam) for 30 minutes at 4°C in the dark, washed twice with ice‐cold PBS and incubated with a secondary FITC‐conjugated antibody (ab6717; Abcam) for 30 minutes at 4°C in the dark. The cells were washed twice with ice‐cold PBS, incubated with 500 µL PBS at room temperature in the dark and examined using flow cytometry.

### 2.3 In vivo and in vitro procedures

#### 2.3.1 Western blot analysis

The total protein from the brain tissue or cells was extracted and quantified using the bicinchoninic acid method. The equivalent weight of the proteins (40 μg/lane) was separated on a 10% SDS‐PAGE gel and was then transferred to a PVDF membrane (Millipore). The membrane was blocked with 5% non‐fat milk in TBST buffer for 1 hour and then incubated overnight with the following primary antibodies: Bax, 2772 (Cell Signalling Technology); Bcl‐2, ab32124 (Cell Signalling Technology); Bcl‐xl, 2764 (Cell Signalling Technology); cleaved caspase‐3, 9664 (Cell Signalling Technology); GAPDH, 2118 (Cell Signalling Technology); and TIM‐4, ab47637 (Abcam). After being washed twice with TBST, the membranes were incubated with a horseradish peroxidase‐conjugated secondary antibody (anti‐rabbit‐HPR; 7074; Cell Signalling Technology) at a 1:2000 dilution. Specific bands were visualized using an enhanced chemiluminescence detection kit.

#### 2.3.2 Real‐time polymerase chain reaction

The total RNA from the brain tissue or cells was extracted and reversed transcribed using TRIzol Reagent (Invitrogen) and a Prime Script reagent RT Kit (Takara Biotechnology) according to the manufacturer's protocol. Real‐time qPCR was performed using an ABI Prism 7900HT Real‐Time System (Applied Biosystems Inc). The following primers were used: IL‐1β, AAATCTCGCAGCAGCACAT (forward) and CACACACCAGCAGGTTATCA (reverse); Cxcl‐1, CTGGGATTCACCTCAAGAACATC (forward) and CAGGGTCAAGGCAAGCCTC (reverse); Cxcl‐2, CCAACCACCAGGCTACAGG (forward) and GCGTCACACTCAAGCTCTG (reverse); TIM‐4, ACACATTTTCCCTGCCTCGT (forward) and GCTGTGGCAAGGATTTCACC(reverse); IL‐6, CCACTTCACAAGTCGGAGGCTTA (forward) and CCAGTTTGGTAGCATCCATCATTTC (reverse); TNF, TATGGCCCAGACCCTCACA (forward) and GGAGTAGACAAGGTACAACCCATC (reverse); and Actin, GACATGGAGAAGATCTGGCACCACA (forward) and ATCTCCTGCTCGAAGTCTAGAGCAA (reverse).

Actin was used as a housekeeping gene. The results were presented as the ratio of the gene to the expression of actin mRNA (sense and antisense).

#### 2.3.3 ELISA

The supernatants were collected and stored at 4°C. Concentrations of LDH, MPO, IL‐6, TNF‐α, IL‐1β, NO and MIP‐1α were detected using commercial ELISA kits (R&D Systems, Inc).

### 2.4 In vitro experiments

#### 2.4.1 Cell culture and TIM‐4 siRNA transfection

Microglia were obtained using a previously described method.^15^ Briefly, primary mixed glial cells were isolated from the cerebrum and cerebellum of mice (1‐day‐old) and placed in six‐well plates at a density of 1.2 × 10^6^ cells/mL of DMEM supplemented with 10% foetal bovine serum (FBS), non‐essential amino acids and insulin. The plates were then placed in a humidified incubator with 5% CO_2_ at 37°C with a medium change every 48 hours. Microglia were isolated from the mixed glial population after reaching confluency (approximately 2 weeks). Microglia cultures with more than 96% purity were used for the study.

Primary neurons were obtained from the cerebral cortex of 1‐ to 3‐day‐old mice Approximately 2 × 10^5^ cells were inoculated to a 25 cm^2^ culture flask pre‐coated with poly‐lysine (the medium was DMEM/F12 high‐glucose medium [containing 1% streptomycin and 20% FBS]). After 24 hours, the medium was changed by Neurobasal Medium containing 1% penicillin (Pen, 100 U/mL) and 1% streptomycin (Strep, 100 U/mL) and a 2% B27 supplement. The neurons were cultured in a humidified incubator with 5% CO_2_ at 37°C. After 6‐7 days of in vitro culture, the cells were examined to ensure >90% purity of neurons, which could be used for further study.

Microglia and neural cells were seeded in Dulbecco's modified Eagle's medium/Nutrient F‐12 Ham containing 10% foetal bovine serum (Gibco) and maintained in a 37°C incubator (Thermo Fisher) in a 5% CO_2_ atmosphere. TIM‐4 siRNA were transfected into microglia for 48 hours using lipofectamine2000 (Invitrogen) in accordance with the manufacturer's protocol. To detect the level of TIM‐4 expression in microglia cells, the cells were stimulated with LPS (100 ng/mL) and IFN‐γ (20 U/mL) for 48 hours and collected for subsequent experiments.

#### 2.4.2 Co‐culture model

Microglia cells were co‐cultured with primary neural cells at a 2:1 ratio for 24 hours. The medium was exchanged with medium containing IFN‐γ (500 U/mL; PeproTech) + LPS (500 ng/mL). TIM‐4 mAb and TIM‐4 mAb + IFN‐γ + LPS group or TIM‐4 siRNA and TIM‐4 siRNA + IFN‐γ + LPS group were incubated with anti‐TIM‐4 (5 ng/mL) antibodies for 2 hours before adding IFN‐γ (500 U/mL) and LPS (500 ng/mL). After 72 hours, photographs were obtained, and the supernatants were collected for LDH measurement.

Microglia cells were co‐cultured with primary neural cells at a 2:1 ratio for 24 hours. The medium was exchanged with medium containing IFN‐γ (20 U/mL; PeproTech) + LPS (100 ng/mL). The TIM‐4 mAb and TIM‐4 mAb + IFN‐γ + LPS group or TIM‐4 siRNA and TIM‐4 siRNA + IFN‐γ + LPS group were incubated with an anti‐TIM‐4 (5 ng/mL) antibody for 2 hours before adding IFN‐γ (20 U/mL) and LPS (100 ng/mL). After 24 hours, photographs were obtained, and the supernatants were collected for an ELISA.

#### 2.4.3 EdU assay

For the EdU staining assay, the cells were permeabilized and stained with a Click‐iT EdU Imaging Kit (Invitrogen) according to manufacturer's instructions.

### 2.5 Statistical analysis

All values are expressed as the mean ± SD. Statistical analysis was performed using Graphpad Prism 5.0 software (GraphPad Software). To compare the difference between two groups or multi‐groups, t test and one‐way ANOVA were performed, respectively. *P* < .05 was considered statistically significant.

## 3 RESULTS

### 3.1 TIM‐4 is positively associated with cerebral IRI in MCAO mice

**Figure 1:**
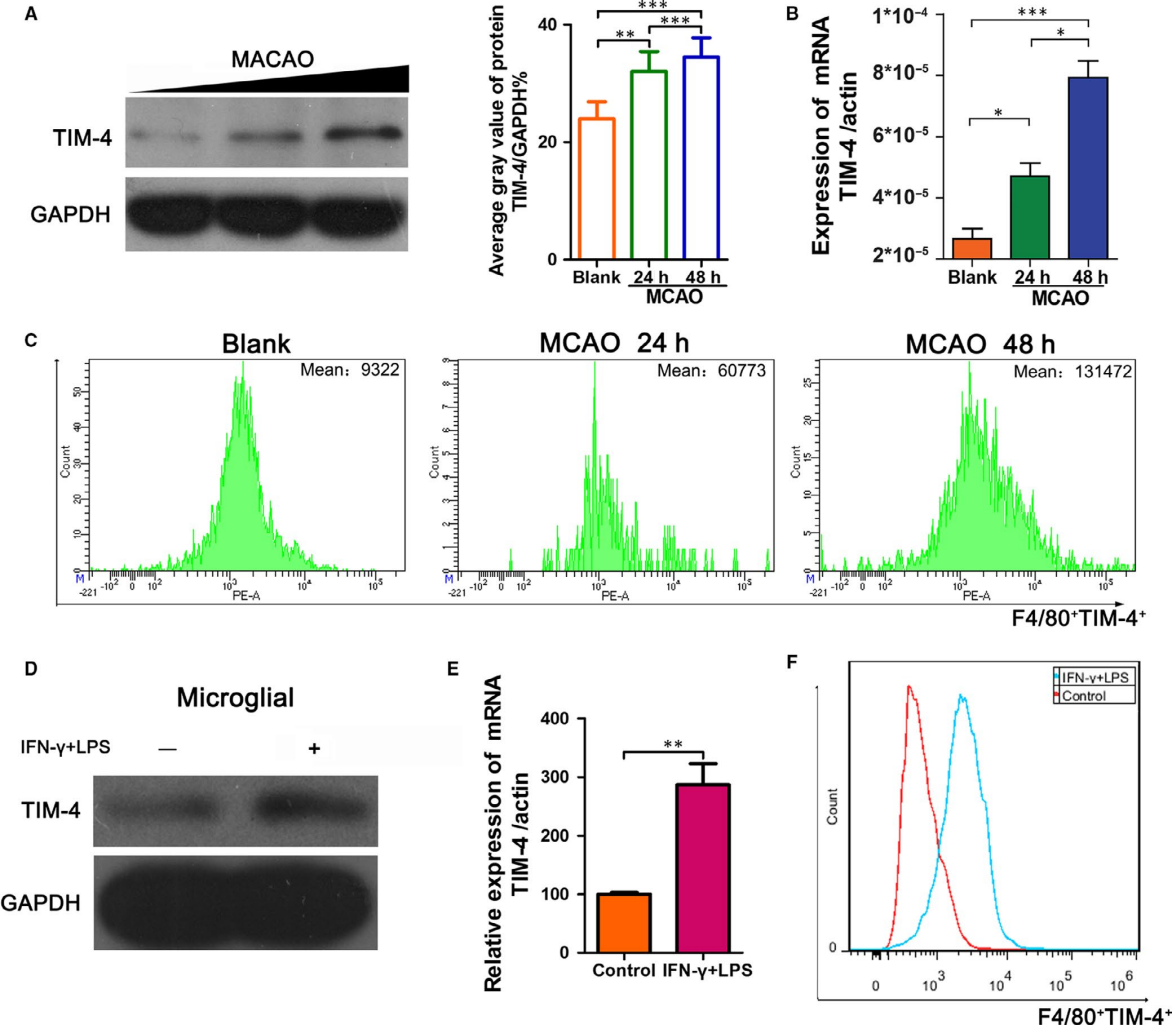
The level of TIM‐4 expression was up‐regulated in MCAO mice. A, Western blot analysis of TIM4 expression at 24 and 48 h after reperfusion. GAPDH was used as the loading control. B, qRT‐PCR analysis of TIM‐4 expression at 24 and 48 h after reperfusion. C, Flow cytometric analysis of TIM4‐positive cells in peripheral blood PBMCs at 24 and 48 h after reperfusion. D, Western blot analysis of TIM‐4 expression after LPS + IFN‐γ stimulation in microglia. GAPDH was used as a loading control. E, qRT‐PCR analysis of TIM‐4 expression after LPS + IFN‐γ stimulation in microglia. F, Flow cytometric analysis of TIM4‐positive cells following the stimulation of microglia with LPS + IFN‐γ (*P < .05; **P < .01; ***P < .001)

To investigate whether TIM‐4 plays a role in cerebral IRI, we established a mouse MCAO model. The mice were divided into three groups: (a) blank, (b) ischaemia for 1 hour and reperfusion for 24 hours and (c) ischaemia for 1 hour and reperfusion for 48 hours. TIM‐4 accumulation and mRNA levels were examined over time in MCAO mice (Figure 1A,B). The Western blot analysis showed that the level of TIM‐4 protein was significantly higher at 48 hours than that at 24 hours after reperfusion, which was consistent with the qRT‐PCR results. We extracted monocytes from the peripheral blood and purified using anti‐CD14 magnetic beads. Flow cytometry showed that the level of TIM4‐positive cells after reperfusion was higher at 48 hours compared with that at 0 hour or 24 hours (Figure 1C). Moreover, the microglia were stimulated with IFN‐γ and LPS in vitro. We examined the expression of TIM‐4 in the control and IFN‐γ + LPS group and found that treatment with IFN‐γ + LPS substantially increased the level of TIM‐4 protein expression and transcription compared to the control group (Figure 1D‐F).

### 3.2 TIM‐4 blockade reduces the infarction area in MCAO mice

**Figure 2:**
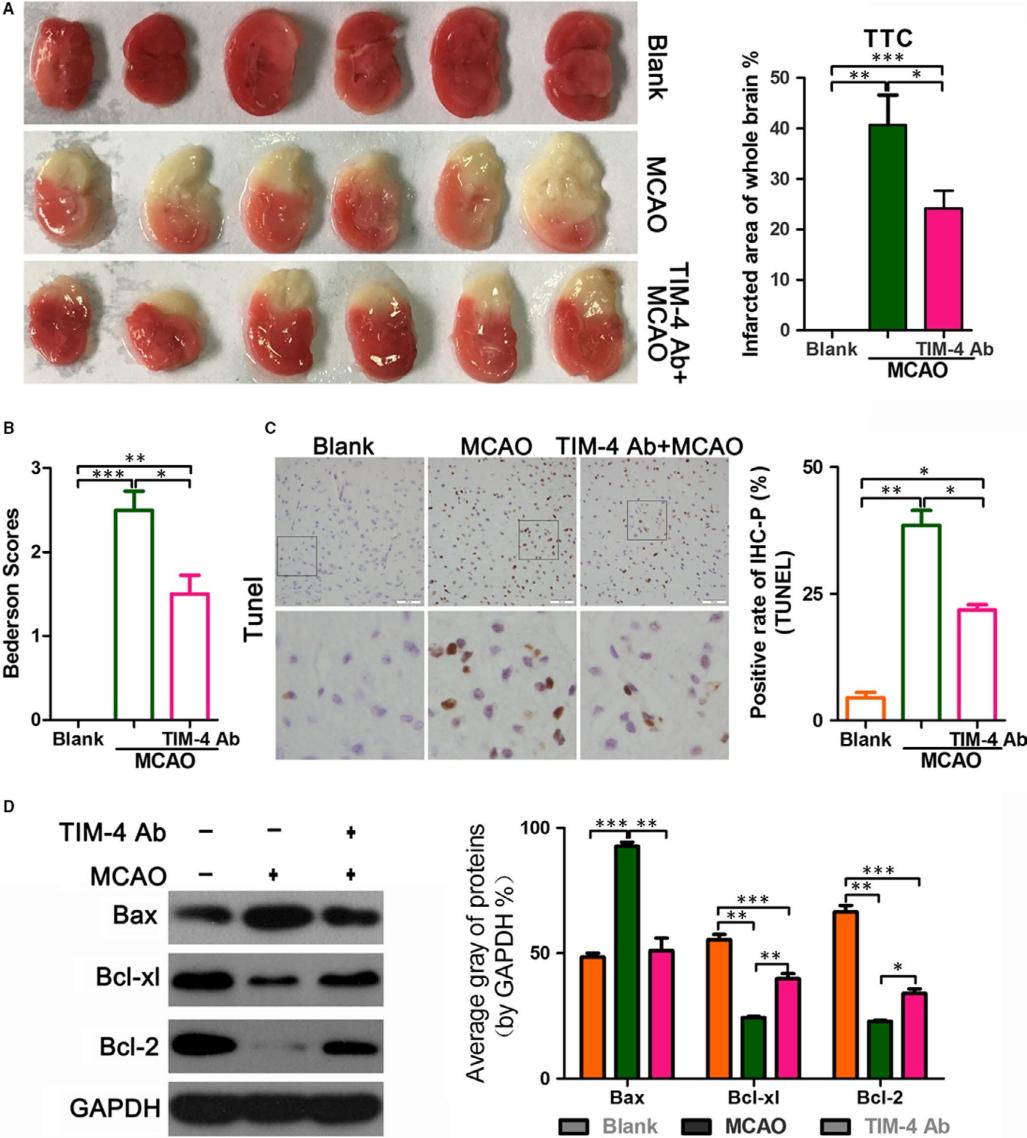
TIM‐4 blocking antibody exerts a protective effect in MCAO mice. Three groups of mice were used in this experiment: (1) untreated sham mice, (2) MCAO mice and (3) MCAO mice treated with an anti‐TIM‐4 antibody for 48 h. A, Representative brain infarction images and quantitative graphs from the three groups of animals. The infarct area was measured using 2,3,5‐Triphenyltetrazolium chloride staining. B, The Bederson score of the three groups of animals. C, Cellular apoptosis was examined using a TdT‐mediated biotin‐16‐dUTP nick‐end labelling (TUNEL) assay. Magnification: ×400. D, Expression of apoptosis‐related proteins measured using a Western blot analysis (*P < .05; **P < .01; ***P < .001)

To further explore the effect of TIM‐4 in cerebral IRI, we tested the therapeutic effect of a TIM‐4 blockade in MCAO mice. There were three groups of mice: (a) a sham blank control, (b) MCAO and (c) MCAO with TIM‐4 mAb administration. TTC staining revealed no colour in the blank group. The infarct area was white in the MCAO group. The infarcted area of whole brain was smaller in the TIM‐4 mAb + MCAO group compared with the MCAO group (Figure 2A). These results suggest that a TIM‐4 blockade reduces the brain infarction area. This TIM‐4 inhibition‐mediated reduction in the infarction area is consistent with the Bederson scores (Figure 2B) since all of the blank mice scored 0, MCAO mice scored roughly 2.5, whereas the anti‐TIM‐4 mAb‐treated mice had an average score of only 1.5. The mice in the MCAO + TIM‐4 mAb group displayed decreased apoptosis compared with that of the MCAO group. The blank‐operated animals exhibited minimal changes. The TUNEL assay showed that MCAO increased cell apoptosis compared with the blank group, whereas TIM‐4 mAb reduced MCAO‐induced apoptosis (Figure 2C). Moreover, the expression of the BCL‐2 and BCL‐XL proteins, which inhibit apoptosis, was higher in the MCAO + TIM‐4 mAb group than in the MCAO group, while expression of the apoptosis promoter BAX protein and the apoptosis marker, cleaved caspase‐3, were lower in the MCAO + TIM‐4 mAb than in the MCAO group (Figure 2D).

### 3.3 A TIM‐4 blockade alleviates the inflammatory response in MCAO mice

**Figure 3:**
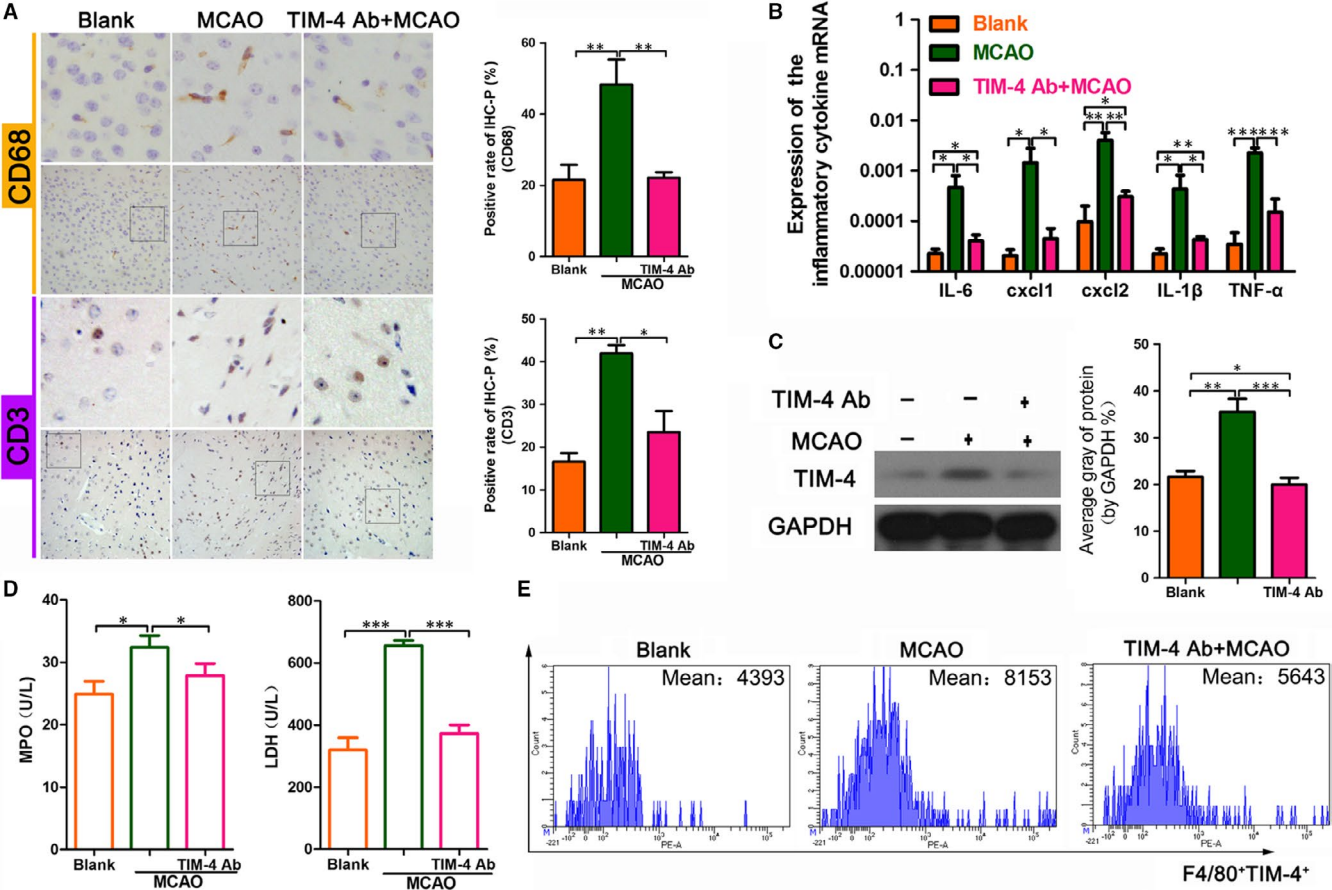
Changes in inflammatory cells and cytokines in MCAO mice treated with or without TIM‐4 mAb. A, CD68‐ and CD3‐positive cells of the three groups of animals on an immunohistochemical analysis. B, Detection of IL‐6, CXCL‐1, CXCL‐2, IL‐1β and TNF‐α using qRT‐PCR in each group. C, Western blot analysis of TIM‐4 expression in each group. D, The concentration of myeloperoxidase (MPO) and lactic dehydrogenase (LDH) was determined using an enzyme‐linked immunosorbent assay. E, Flow cytometric analysis of TIM4‐positive cells in peripheral blood PBMCs. (*P < .05; **P < .01; ***P < .001)

To further explore the potential contribution of the TIM‐4 blockade in MCAO mice, we next examined the level of inflammatory cells and cytokines in each of the groups. The immunohistochemical analysis showed that the level of the macrophage marker, CD68, and the T cell marker, CD3, was lower in the MCAO + TIM‐4 mAb group than in the MCAO group, which indicated less macrophage and T cell infiltration in the former group (Figure 3A). The qRT‐PCR assay showed that the expressions of the inflammatory cytokines IL‐6, CXCL‐1, CXCL‐2, IL‐1β and TNF‐α were lower in the MCAO + TIM‐4 mAb group than in the MCAO group (Figure 3B). The Western blot analysis showed that the level of TIM‐4 protein in the MCAO + TIM‐4 mAb group was lower than that in the MCAO group (Figure 3C). Additionally, the myeloperoxidase (MPO) and lactic dehydrogenase (LDH) concentration were lower in the MCAO + TIM‐4 mAb group than in the MCAO group (Figure 3D). The flow cytometric analysis showed that the number of TIM‐4‐positive cells was decreased in the MCAO + TIM‐4 mAb group compared with that in the MCAO group (Figure 3E).

### 3.4 The damaging effect of microglia on the neurons was significantly reduced after an anti‐TIM‐4 antibody blockade

**Figure 4:**
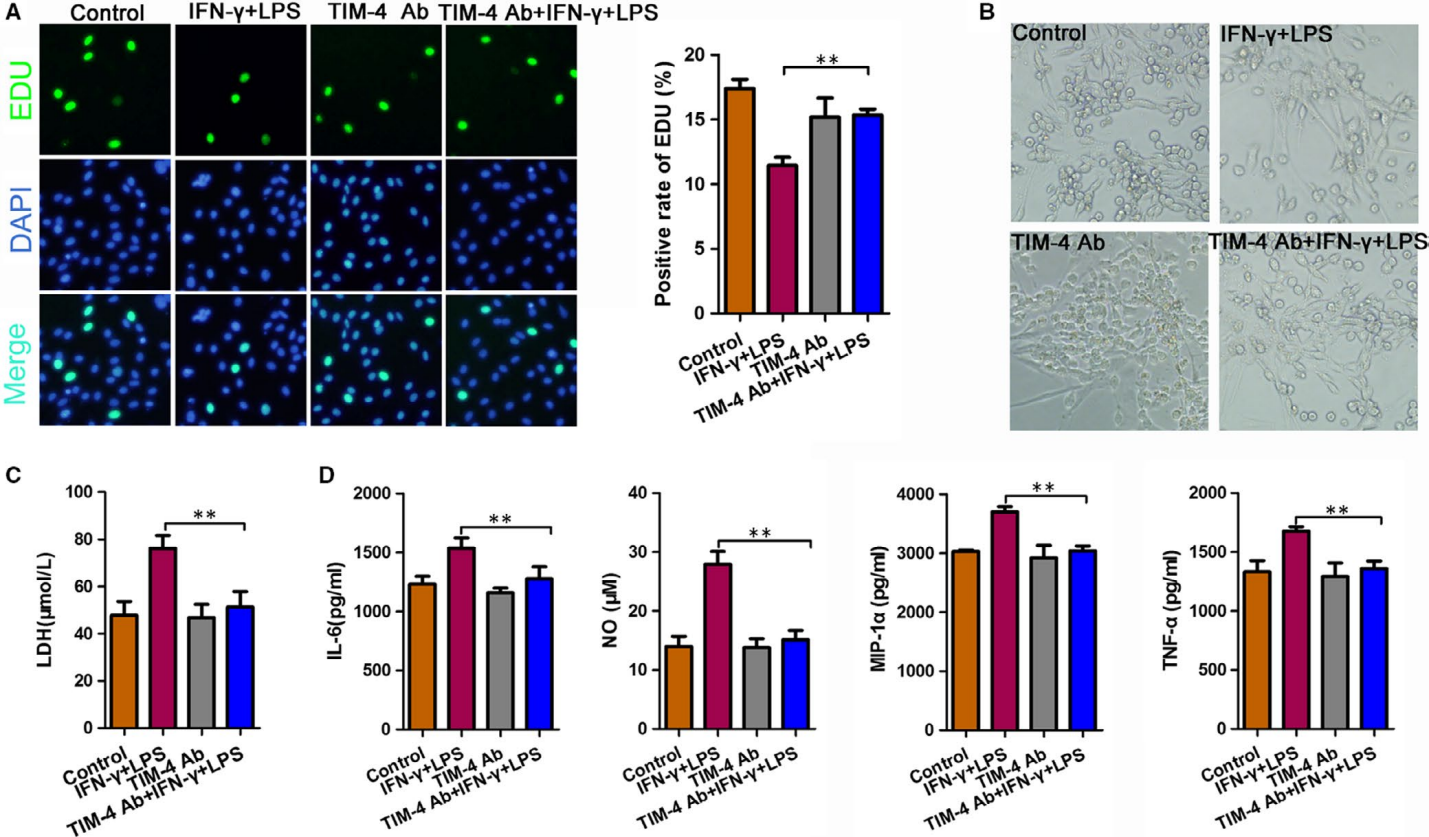
Microglia‐mediated neuronal damage in microglia co‐cultured with neurons following an anti‐TIM‐4 antibody blockade. A, An EdU assay showed the positive rate of EdU changes when the microglia cells were co‐cultured with neurons for 24 h under different conditions. B, Bright field microscopy showing the morphological changes that occurred when the microglia cells were co‐cultured with neurons for 24 h under different conditions. C, Lactic dehydrogenase (LDH) concentration determined using an enzyme‐linked immunosorbent assay. D, The concentration of IL‐6, NO, MIP‐1α and TNF‐α was determined using an enzyme‐linked immunosorbent assay. (**P < .01)

To identify the effect of TIM‐4 on neurons, we co‐cultured microglia cells with nerve cells and then stimulated the cells with LPS + IFN‐α. The TIM‐4 mAb group and TIM‐4 mAb + IFN‐γ + LPS group were incubated with anti‐TIM‐4 (5 ng/mL) antibodies for 2 hours before stimulation with IFN‐γ and LPS. The EdU assay indicated that LPS + IFN‐α stimulation decreased the positive rate of EdU as compared to the negative control, which was prevented by treatment with an anti‐TIM‐4 antibody (Figure 4A). We used light microscopy to detect the morphology of the total neuronal cells in each treatment group (Figure 4B). Moreover, an ELISA showed that the expression of LDH in the LPS + IFN‐α group was significantly higher than that in the NC group, indicating worse damage was observed in the LPS + IFN‐α group. While treatment with the anti‐TIM‐4 antibodies combined with LPS + IFN‐α decreased LDH expression compared to the LPS + IFN‐α group (Figure 4C). In addition, the expression of TNF‐α, IL‐6, MIP‐1α and NO was significantly increased in the LPS + IFN‐α group compared with that in the NC group. However, these levels were decreased following the TIM‐4 blockade (Figure 4D).

### 3.5 The effect of microglia‐mediated damage on neurons was significantly reduced after a TIM‐4 knockdown

**Figure 5:**
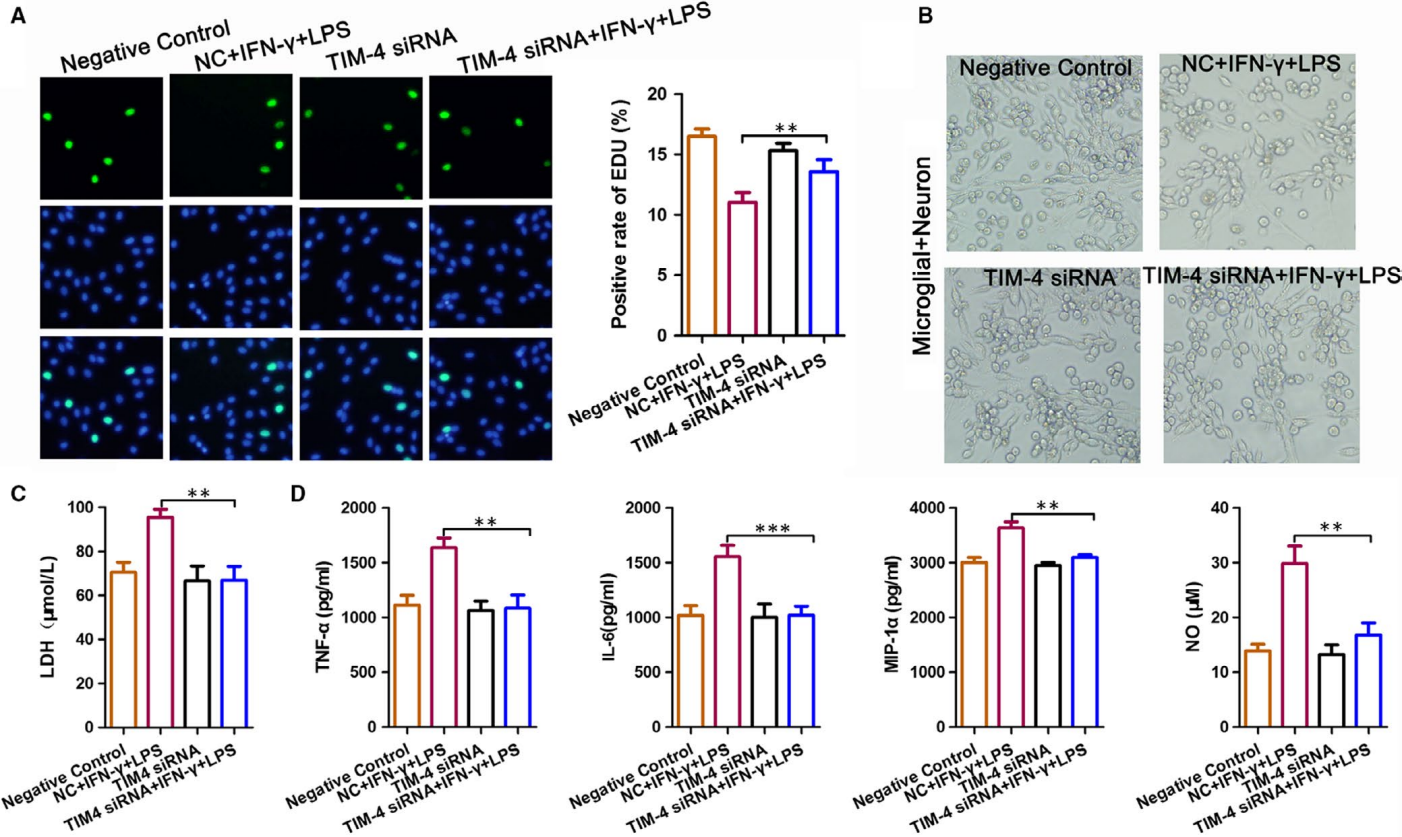
Microglia‐mediated neuronal damage in microglia co‐cultured with neurons following a knockdown of TIM‐4. A, EdU assay showed a positive rate of EdU changes when microglia cells TIM‐4 knockdown were co‐cultured with neurons for 24 h in different conditions. B, Bright field microscopy showing the morphological changes that occurred when TIM‐4 was knocked down in microglia cells co‐cultured with neurons for 24 h under different conditions. C, Lactic dehydrogenase (LDH) concentration determined using an enzyme‐linked immunosorbent assay. D, The concentration of IL‐6, NO, MIP‐1α and TNF‐α was determined using an enzyme‐linked immunosorbent assay (**P < .01; ***P < .001)

To determine the effect of TIM‐4‐mediated damage on neurons, microglial cells were transfected with TIM‐4 siRNA, co‐cultured with nerve cells and stimulated with LPS + IFN‐α. An EdU assay indicated that stimulation with LPS + IFN‐α decreased the positive rate of EdU compared to the control, which was prevented by transfection with TIM‐4 siRNA (Figure 5A). We used light microscopy to detect the morphology of the total neuronal cells in each treatment group (Figure 5B). Moreover, an ELISA revealed that the expression of LDH in the LPS + IFN‐α group was significantly higher than that in the NC group, indicating that worse damage occurred in the LPS + IFN‐α group. The cells transfected with TIM‐4 siRNA prior to LPS + IFN‐α prevented the LPS + IFN‐α ‐induced damage (Figure 5C). In addition, the expression of TNF‐α, IL‐6, MIP‐1α and NO was significantly increased in the LPS + IFN‐α group compared with the NC group. However, these levels were decreased after transfection with TIM‐4 siRNA (Figure 5D).

## 4 DISCUSSION

Ischaemic stroke is one of the leading causes of morbidity and mortality globally and is a major cause of long‐term disability in both developed and developing countries.^16^ Insufficient cerebral blood flow can cause hypoxia, inflammation, oxidative stress, glutamate excitatory toxicity and blood flow reperfusion can aggravate these symptoms, a process termed IRI.^17^ Following cerebral I/R, inflammation induces the infiltration of peripheral inflammatory cells, microglia activation and the over‐generation of inflammatory mediators (eg cytokines), which causes immune activation and secondary ischaemic injury to the brain tissue.^18^ The inflammatory reaction and apoptosis play an important role in cerebral IRI and are the main factors inducing nerve cell injury following IRI.^19^ Therefore, reducing inflammation is currently the primarily goal of many research laboratories worldwide.

In the present study, the role of TIM‐4 in IRI was investigated. TIM‐4 is located on chromosome 5q23, primarily expressed on macrophages, mature dendritic cells and some peritoneal B cells but not T cells, and is a member of the T cell immunoglobulin domain and mucin domain gene family that plays a critical role in regulating the immune response.^20^, ^21^, ^22^ TIM‐4 was initially identified as a phosphatidylserine (PS) ligand receptor capable of binding/engulfing apoptotic bodies, the key phagocytosis step in innate immune reactions.^21^ The function of TIM‐4 in the immune response has been viewed largely through this paradigm.^20^, ^23^ However, the precise role of TIM‐4 has been complicated by contradictory findings. TIM‐4 was first believed to promote T cell proliferation by interacting with TIM‐1, a costimulatory molecule expressed on activated T cells.^20^, ^24^ Later, the interaction between TIM‐1 and TIM‐4 was shown to occur via bridging exosomes.^25^ Subsequently, TIM‐4 was shown to bind to an unknown inhibitory ligand on naive T cells.^24^ These findings suggest that TIM‐4 inhibits naive immune responses but promotes effector responses.

Accumulating evidence has shown that the TIM‐4 pathway plays an important role in IRI.^10^, ^12^, ^22^ TIM‐4 expression is also increased in ischaemic stroke patients.^26^ Thus, we hypothesized that TIM‐4 might participate in cerebral IRI. To test this hypothesis, we established a MCAO model. The Western blot and qRT‐PCR assays showed that the level of TIM‐4 expression was increased following reperfusion. Moreover, the number of TIM‐4‐expressing monocytes separated from PBMCs was increased following reperfusion. These data suggest that TIM‐4 expression is increased in cerebral IRI, indicating that IRI induced the up‐regulation of TIM‐4. Next, to detect the effect of TIM‐4, we used a mouse model treated with anti‐TIM‐4 antibody. TTC staining revealed the infarcted area of the whole brain was smaller in the TIM‐4 mAb + MCAO group compared with the MCAO group. A TUNEL assay showed that MCAO was associated with increased cellular apoptosis, whereas treatment with an anti‐TIM‐4 antibody reduced MCAO‐induced apoptosis. This was consistent with the Western blot results. The Western blot showed that the apoptosis inhibitors, BCL‐2 and BCL‐XL proteins, were higher, whereas the apoptosis promoter, BAX protein, and the apoptosis marker, cleaved caspase‐3, were lower in the MCAO + TIM‐4 mAb compared to that of the MCAO group. These results illustrate that the TIM‐4 blockade reduced IRI‐induced damage and apoptosis.

Accumulating evidence shows that ischaemic injury and inflammation account for the pathogenic progression of stroke.^18^, ^27^ In the present study, we found that the level of the macrophage marker, CD68, and T cell marker, CD3, expression were lower in the MCAO + TIM‐4 mAb group compared with that in the MCAO group, indicating that TIM‐4 mAb reduced macrophage and T cell infiltration. The inflammatory cytokines IL‐6, CXCL‐1, CXCL‐2, IL‐1β and TNF‐α were lower in the MCAO + TIM‐4 mAb group compared with that in the MCAO group, suggesting that inhibiting TIM‐4 reduced inflammatory cytokine production. In addition, the level of myeloperoxidase and lactic dehydrogenase concentration was lower, and the number of TIM‐4‐positive cells was decreased in the MCAO + TIM‐4 mAb group compared with that in the MCAO group.

Monocytes are important inflammatory cells, which play an antigen‐presenting role in the acquired immune response and are the main innate immune receptors. Under stimulation with various factors, monocytes can secrete several cytokines (eg IL‐1, IL‐6, IL‐8, IL‐10 and TNF‐α). It is known that stimulation with LPS or IFN‐γ induces TIM‐4 expression in macrophages.^28^ In the study by Rodriguez‐Manzanet,^29^ activated macrophages induced by LPS were found to up‐regulate TIM‐4 expression. This finding is consistent with our finding that the expression of TIM‐4 was higher following stimulation of microglia with LPS + IFN‐γ. Next, we co‐cultured microglia with primary nerve cells to observe the effect of TIM‐4 on neurons. We first divided the co‐cultured cells into the control, LPS + IFN‐γ, TIM‐4 mAb and LPS + IFN‐γ + TIM‐4 mAb groups. Treatment with LPS + IFN‐γ decreased the level of cellular proliferation, whereas treatment with LPS + IFN‐γ + TIM‐4 mAb reversed this effect. Following stimulation with LPS + IFN‐γ, LDH, and cytokines (eg IL‐6, NO, TNF‐α, and MIP‐1α) was increased while the administration of TIM‐4 mAb had the opposite effect. These results show that the inhibition of TIM‐4 alleviates the damage of TIM‐4 on neurons. The microglia were transfected with TIM‐4 siRNA and subsequently co‐cultured with primary nerve cells, followed by stimulation with LPS + IFN‐γ. We found that the knockdown of TIM‐4 alleviated the damage of TIM‐4 on neurons.

Although we have shown in mice that inhibiting TIM4 protects against reperfusion injury, there are still many limitations of our study. Firstly, there are some fundamental differences in cerebral perfusion between rodent models and human brains. Secondly, in order to avoid the interference of other physiological factors, most of the animal models adopted younger mice. But that does not work in human patients because human get older and the timing of the disease unpredictable. Thirdly, in order to build the MCAO model, we would inject chloral hydrate intraperitoneally and anesthetize the mice. It does not work with human patients either. Finally, we can artificially control the onset time of animals in animal models and inject TIM4 antibody in advance. But timing of investigational product infusion is mostly not feasible in humans.

In summary, the findings of our study demonstrate that cerebral IRI up‐regulates the expression of TIM‐4, and the inhibition of TIM‐4 markedly decreased the cerebral infarction area, reduced apoptosis and the number of inflammatory cells after MCAO. In vitro inhibition of TIM‐4 in activated microglia treated with anti‐TIM‐4 antibodies or transfected with TIM‐4 siRNA alleviated TIM‐4‐mediated damage to nerve cells. Therefore, these findings suggest an important role for TIM‐4 in cerebral IRI and co‐cultured cells, indicating a potential target for the treatment of ischaemic stroke.

## CONFLICT OF INTEREST

The authors declare that they have no competing interests.

## AUTHOR CONTRIBUTIONS

PCL and CW conceived the research idea; HYQ and WXH performed the experiments; WXJ and CW analysed the data; and ZLF wrote the manuscript. All authors read and approved the final version of the manuscript.
